# Extraction of 2′-*O*-apiosyl-6′-*O*-crotonic acid-betanin from the ayrampo seed (Opuntia soehrensii) cuticle and its use as an emitting layer in an organic light-emitting diode

**DOI:** 10.1039/d0ra05543c

**Published:** 2020-10-06

**Authors:** Harry Anderson Rivera Tito, Gerardo Hernández-Sosa, Carlos Romero-Nieto, Elzbieta Regulska, Nils Jürgensen, Johannes Zimmermann, Karim Salazar-Salinas, María Esther Quintana Caceda

**Affiliations:** Center for the Development of Advanced Materials and Nanotechnology, National University of Engineering Av. Tupac Amaru 210 Lima 25 Peru mquintana@uni.edu.pe; Light Technology Institute, Karlsruhe Institute of Technology Engesserstr. 13 76131 Karlsruhe Germany; Innovation Lab Speyerer Straße 4 69115 Heidelberg Germany; Organic Chemistry Faculty, Heidelberg University Grabengasse 1 69117 Heidelberg Germany; Faculty of Chemistry, University of Bialystok Ciolkowskiego 1K 15-245 Bialystok Poland; Environmental Engineering Department, Cayetano Heredia University Av. Honorio Delgado 430, Urb. Ingeniería, S.M.P Lima 15102 Peru

## Abstract

The molecule 2′-*O*-apiosyl-6′-*O*-crotonic acid-betanin (called Achkiy) was obtained after an ecofriendly and low-cost purification process of the extract from the ayrampo seed cuticle. Results from EDS give us an idea of the organic elements present in the ayrampo cuticle layer composed of carbon, oxygen and nitrogen. Further characterization analysis of ayrampo extract by Fourier Transform Infrared Spectrophotometry (FTIR) corroborated the presence of characteristic functional groups corresponding to carboxyl, carbonyls, hydroxyls and secondary amines. On the other hand, we have confirmed by absortion peak the glucose, apiosyl, crotonic acid and betanin at 227 nm, 276 nm, 291 nm and 534 nm bands respectively. Mass Spectrometry (MS) characterization was used finally to identify the electroactive Achkiy molecule. This molecule was tested in an Organic Light Emitting Diode (OLED) achieving a luminance of 4.8 Cd m^−2^ when bias voltage of 16.5 V and a current of 34.1 mA was applied. In addition, the irradiance generated by the Achkiy layer reaches a value of ≈ 113.3 μW m^−2^ emitting light with a *λ* ≈ 390.10 nm. These preliminary results report an interesting molecule extracted from a natural pigment wich emits light in the blue region.

## Abstract 2

The molecule 2′-*O*-apiosyl-6′-*O*-crotonic acid-betanin (called Achkiy) was obtained after an ecofriendly and low-cost purification process of the extract from the ayrampo seed cuticle.

## Graphical abstract

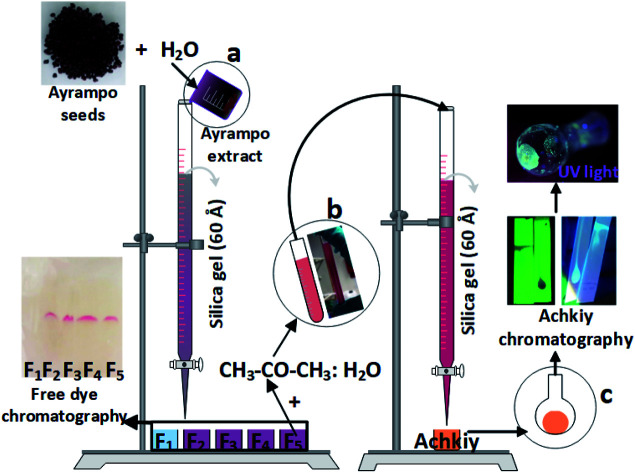

## Introduction

The importance of incorporating eco-friendly materials in light-emitting devices has led to a continuous search for new electroactive materials from natural sources. These materials require specific characteristics, which are tunable to manufacture inorganic and organic diodes and electrochemical light emitting cells (ILEDs, OLEDs and LECs) by common coating techniques such as inkjet printing, doctor blade and spin coating.^1–4^ For example, one of the natural sources comes from plant extracts, which have been used in electronic devices as photo sensitizing materials^5–7^ and colorimetric indicators in sensors.^8^ The identification of electroactive components from different plant parts (leaves, seeds, flowers, pulp, peel) is relevant to develop plant-based materials and bio-optoelectronic devices. In order to isolate these components from the plant extract, studies suggest using High-Performance Liquid Chromatography (HPLC) techniques, which is an expensive technique^9,10^ As well, riboflavin obtained from B_2_ vitamin has been reported as an electroluminescent layer in an OLED device. The performance of this device reached a luminance of 10 Cd m^−2^ emitting light at 640 nm corresponding to the orange region.^4^

The betalain is a huge group of plant pigments, which chemically corresponds to conjugates of the betalamic acid chromophore that derives from 3-(3,4-di hydroxyphenyl) alanine (DOPA) *via* oxidative mechanism of 4,5-extradiol ring opening. This group has two subcategories: betacyanins (red color) and betaxanthins (yellow color). The betanins belong to the betacyanin subcategory and their base core structure is derived from betanidin. The betanin family structure has two radicals R1 and R2 at carbon 5 and 6, respectively, through the OH molecule, where the radical R1 is β-d-glucose molecule and R2 is OH. The replacements of the radicals cause the great variety of betanin pigments with different photoluminescence.^11,12^

The photochemical activity of betacyanins subcategory brings out the *λ* absorption at green region. The use of betacyanins (betanidin and betanin) in photovoltaic devices has been reported in the literature taking in advantage its photoactivity.^5,6^ The purified betanin emits cold light by the photoluminescence process, which its emission peak corresponds to 608 nm when it is excited with an incident light wavelength of 535 nm. While, betaxanthin subcategory, such as indicaxanthin, has a higher value.^11^ The ayrampo is an Andean plant (Opuntia soehrensii), and its seed pigments have been used to dye food,^13^ textile and ceramic handcraft^14,15^ because of its intense purple-red color. In the following paper, we are developing an experimental line to isolate photoelectric active molecules from ayrampo. This procedure is economically affordable and green production approach to produce eco-friendly materials without using HPLC technique. The follow-up purification processes are tuned by characterization techniques such as SEM microscopy, X-ray Energy Dispersion Spectrometry (EDS), Fourier-Transform Infrared Spectroscopy (FTIR) and UV-vis. In addition, we use the advanced Mass Spectroscopy (MS) technique to analyse the molecular weight of the extracted pigment and identify the photo active pigment compound, which we call Achkiy. Afterwards, the purified molecule was used to prepare a light emitter layer in an OLED flat electroluminescent device, which the superimposed layers is glass/ITO/Achkiy/TPBi/LiF/Aluminium and it was compared with the betanin layer analog. Our results suggest that Achkiy molecules presents electroluminescent effect because of their structural basis.

## Materials and methods

### Software

*Molinspiration* software calculates the octanol–water partition coefficient (logP) by mathematical method of the miLogP software from the atomic hydrophobia contributions of fragments. These have been obtained by fitting calculated logP with experimental logP for a training set more than twelve thousand, mostly drug-like molecules. In this way hydrophobicity values for 35 small simple “basic” fragments have been obtained, as well as values for 185 larger fragments, characterizing intramolecular hydrogen bonding contribution to logP and charge interactions. The logP value is used as a measure of molecular hydrophobicity.

*General Atomic and Molecular Electronic Structure System (GAMESS)* software uses the method self-consistent field restricted Hartree–Fock (RHF) in order to optimize the proposed structure of the Achkiy pigment under vacuum condition. The frontier molecular orbital, that is, the highest occupied molecular orbital (HOMO) and the lowest unoccupied molecular orbital (LUMO), are important indicators to understand the charge transfer.^16^

### Ayrampo seeds conservation and characterization

#### Procedure

Ayrampo seeds were bought in the main market of Cusco city – Peru. They were transported in dark bags, made of newspaper, to prevent contamination and discoloration. To preserve the samples, they were stored in a refrigerator at 2 °C with a relative humidity of less than 30%. Afterwards ayrampo seeds were characterized using a Field Emission Scanning Electron Microscope (FE-SEM) HITACHI model SU823O.

### Extraction of Achkiy from ayrampo seeds

#### Materials

The acetone (99.6% purity) was acquired from the company J. T. Baker, the ultrapure water was collected from a Millipore Simplicity 185 water purifying system and the citric acid, anhydrous (MW = 192.12) was purchased from Sigma Aldrich. Moreover, the silica gel (60 Å) was purchased from Fluorochem Ltd. UK. The paper for silica chromatography was provided by the organic chemistry laboratory of the University of Heidelberg. Likewise, we characterize the elemental composition of the extracted pigment by the X-ray Energy Dispersive Spectroscopy (EDS) using a Bruker multichannel detector model Xflash FladQUAD 5060F.

To detect the different functional groups in the Ayrampo extract, we used an infrared spectrophotometry unit with Fourier transform (FTIR) Thermo Fisher Scientific model Nicolet iS10 FT-IR spectrometer. The following coloured fractions obtained through the liquid column chromatography stationary process were characterized by a UV-vis spectrometer model USB 4000 brand Ocean Optics, which consists of two lamps (deuterium and halogen) with electromagnetic wave range from 300 nm to 800 nm.

Finally, we used a HEROLAB UV-8 S/L lamp KW 254 nm and LW 365 nm for the photoluminescence detection of the selected column chromatography fraction.

#### Procedure

**Fig. 1:**
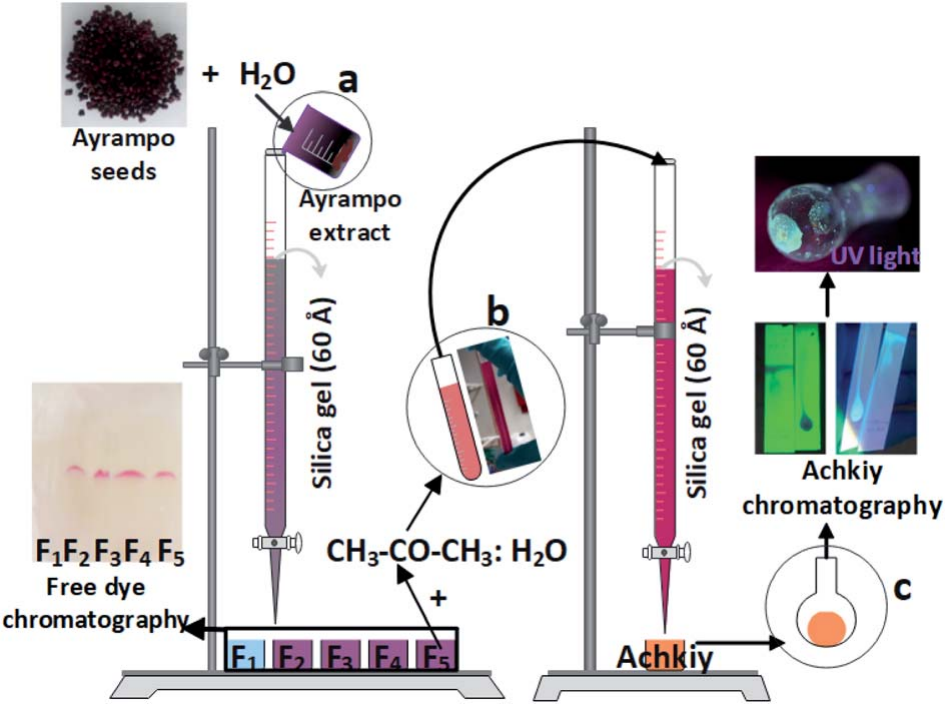
Scheme to obtain Achkiy. (a) Collection of colored ayrampo fractions diluted in water through the chromatography column. (b) Mixture of fraction five in CH_3_–CO–CH_3_ : H_2_O and the separation of the photoluminescent fraction. (c) Use of the silica paper to analyze the photoluminescent fraction.

To extract the pigment contained in the ayrampo cuticle, we weight 5 g of ayrampo seeds on a clock glass to then dry them in an oven at 60 °C overnight. After that, we weight 4 g of the dried seeds in a 50 mL beaker, and add 20 mL of ultra-pure water. The mixture was taken to a shaker (Daihan Sho, digital orbital shaker, SHO-2D) for 4 h at a speed of 150 rpm at room temperature. Then the mixture was filtered using Whatman paper filter grade 41, and then we took 5 mL of this solution to a centrifuge at 6000 rpm for 20 min. The pigment was collected at the bottom of the conic tube, and then dried in the oven for 6 h at 50 °C.^17^ The dried sample was diluted in 15 mL of ultrapure water and purified by chromatography column, using silica gel powder as stationary phase, reaching five fractions with different colours (Fig. 1a). These fractions were further analyzed by Thin Layer Chromatography (TLC) and then the solvents were removed using a rotary evaporator at 90 rpm, 40 °C, with a vacuum of 1066 psia. All the fractions were characterized by UV-vis spectroscopy to select the fraction for the following separation process. According to the results. The fraction 5 was selected to continue the separation of the current photoluminescent compound because this fraction showed the absorption band more well-defined than the others. The selected fraction was diluted with hydrated acetone at pH = 4.6, due to its viscous texture. To further separate it, we were used TLC, and its solvent was extracted using a rotary evaporator at 40 °C, with a vacuum of 1066 psia.

### Achkiy characterization

The analysis of the *m*/*z* ratio molecule contained in the extracted photoluminescent fraction was characterized by a Bruker brand Mass Spectrometer (MS) model Apex-Qe.

### OLED fabrication

#### Materials

The control sample of betanin for the experiments was purchased from Tokyo Chemical Industry Co., LTD. The acetone (99.8%) was purchased from the Carl Roth Company. The electronic grade glass coated with Indium Tin Oxide (ITO) (180 nm, 10 Ω □^−1^, work function indicated by the supplier) was purchased from Kintec, which was used as a Hole Transport Layer (HTL). Lithium Fluoride (LiF) and 2,2′,2′′-(1,3,5-benzinetriyl)-tris(1-phenyl-1-H-benzimidazole) (TPBi) were purchased from Sigma Aldrich and their parameter of work functions were indicated in the product by the supplier. LiF works as Electron Injection Layer (EIL) and TPBi as the Electron Transport Layer (ETL), respectively and the aluminium pellets were acquired from the company Heraeus Group.

#### Procedure

We manufacture an OLED device with an active area of 24 mm^2^ by the following procedure. First, we wash the commercial ITO coated substrate with acetone solution for 10 minutes followed by the 2-isopropanol washing, both using an ultrasonic bath, to remove impurities. Further clean process is performed using O_2_ plasma (Electric plasma surface technology model LFG 40) to remove microbiological contaminants. For then, to deposit the Achkiy thin layer onto ITO coated glass. To accomplish it, we dilute 37.17 mg of Achkiy in 1.2 mL of hydrated acetone (CH_3_–CO–CH_3_ : H_2_O, 8 : 1 ratio), and stir the solution for 3 hours until the Achkiy is completely diluted using a shaker. After this process, Achkiy's thin films were manufactured using a spin coater (KIT model MCD200-NPP). Table 1 shows the setting parameters to obtain the thin film: spin speed (*ω*), spin time (*t*), volume of solution (μL) and drying temperature (*T*).

**Table 1:** Parameters for Achkiy thin film electroluminescent layer production onto glass/ITO

Fraction	*ω* (rpm)	*t* (s)	μL	*T* (°C)
F1	3500	10	210	100

The glass/ITO/Achkiy samples are transferred into a glovebox integrated with a thermal evaporation system to deposit the TPBi thin layer of 45 nm through a shadow mask under a vacuum (working at 10^−6^ mbar). The subsequent thin layer of LiF (1 nm) was deposited onto glass/ITO/Achkiy/TPBi. Finally, the glass/ITO/Achkiy/TPBi/LIF was sealing with a thin layer of Aluminium (70 nm).

### OLED device characterization

We characterize the Achkiy thin layer thickness using a Veeco DEKTAK 150 profilometer. The morphology and topography of the Achkiy layer was characterized using photoluminescence microscopy (Nikon Eclipse 80i Microscope with: LU Plan Fluor 50X objective, Intensilight C-HGFI Precentered Fiber Illuminator with an 380–600 nm mercury lamp, and an EPI-FL B 2E/C filter), Scanning Electron Microscopy (ZEISS scanning electron microscope model Cross Beam Auriga) and Atomic Force Microscopy (DME DualScope DS 95-50E atomic force microscope). Further, we perform Luminance–Current–Voltage (LIV) characterizations of the OLED device by using a Botest LIV functionality test system, calibrated inside the glovebox compartment. The LIV characterizations were measured with a sweeping rate of 200 mV s^−1^ and the electroluminescence was characterized in a glovebox using a USB2000 + UV-vis brand spectrometer Ocean Optics.

## Results and discussion

**Fig. 2:**
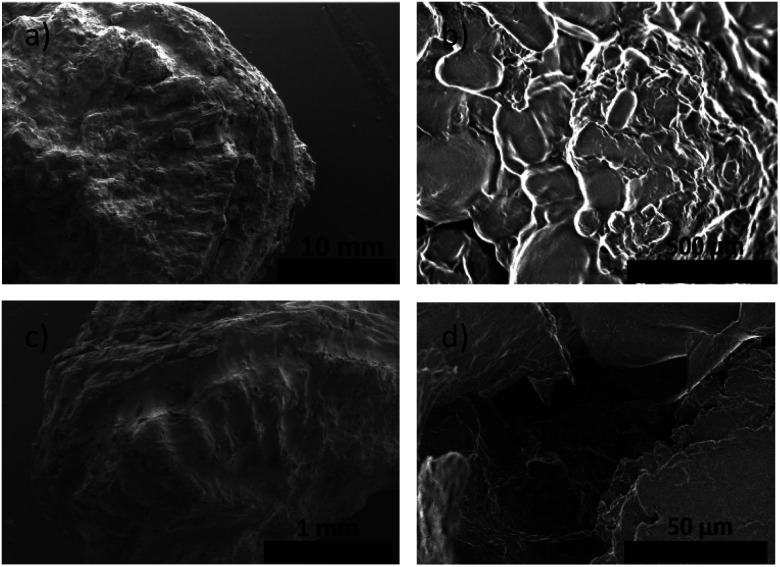
(a) SEM images of the ayrampo seed surface. (b) The cuticle surface, which is the place where the pigment is stored. (c) Hard lignin shell that surrounds the seed embryo, layer seen after be extracted the cuticle. (d) Zoomed in on hard lignin shell micro cavities.

The ayrampo seed is composed by different layered materials; we characterized the surface of these layers using Field Emission Electron Scanning Microscopy (FE-SEM) microscopy in order to evaluate the presence of the pigment layer and its efficient removal. Our results show that the outermost layer of the ayrampo seed (the cuticle) contains almost all the pigment. While, the hard lignin layer does not. Fig. 2 shows the images of the ayrampo seeds before and after the cuticle extraction using deionized water. The difference of the images between Fig. 2a and c reveals that almost all the cuticle was successfully extracted. Likewise, after the cuticle removal process it was observed that the hard lignin layer presents micro cavities, and flat and amorphous surface spaces (Fig. 2d). These micro cavities could create large surface area to absorb water and nutrients from the soil. Since, the plant germinates in rocky and moist soils of the Andes.

**Fig. 3:**
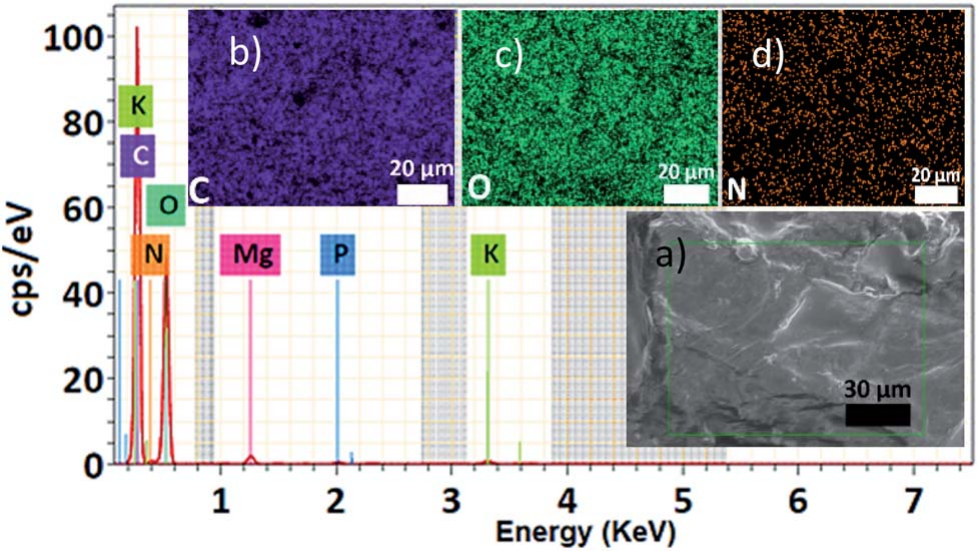
Ayrampo pigment EDS spectroscopy, energy peaks of each element with its corresponding cps eV^−1^. (a) SEM of the ayrampo cuticle surface, where each element is represented by a color, the most abundant being C (b), O (c) and N (d).

The free dye into the ayrampo extract was obtained from the cuticle and we run X-ray Energy Dispersion (EDS) analysis to study the composition. Fig. 3 shows a cluster of elements in the 120 μm × 100 μm surface. In the inset 3(a) we appreciate a caramel-like texture. The energy peaks from the kilo electronvolts (keV) *vs.* counts per second per electronvolt (cps eV^−1^) graph show that the chemical elements contained in the ayrampo pigment are mostly made up of the carbon elements (49.65%), oxygen (44.64%) and nitrogen (3.16%). Moreover, the trace elements are magnesium (0.67%), potassium (1.56%) and phosphorus (0.31%). Corroborated that pigments with photoluminescence response from this source is heavy metal free. The low percentage of the K and Mg contained into extract suggest that possibly the pigment presents negative charged functional groups able to drag the ions by electrostatic interaction. Furthermore, insets b–d suggest us the hydrophilicity of the pigments.

**Fig. 4:**
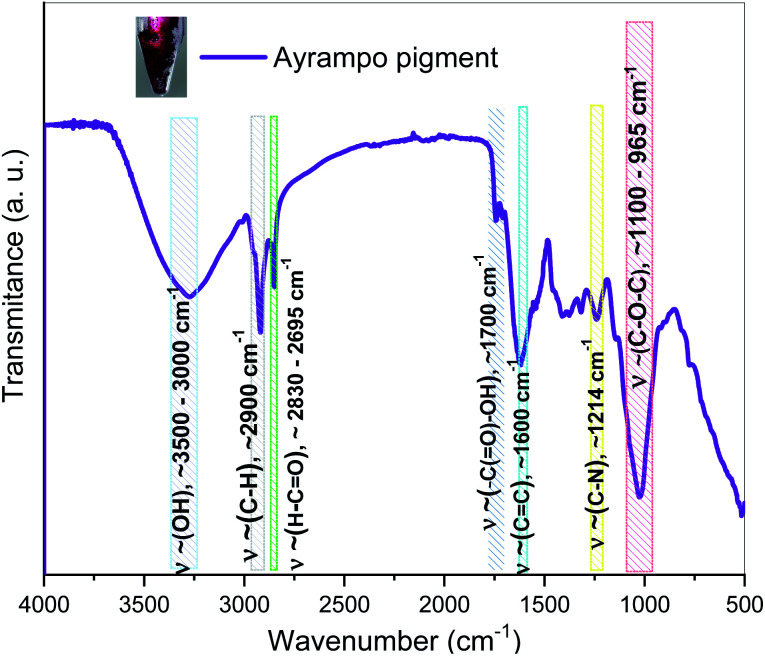
FTIR spectroscopy of ayrampo pigment contained into the extract. Coloured regions represent the vibrational bands corresponding to functional groups.

On the other hand, Fourier Transform Infrared Spectrophotometry (FTIR) spectroscopy in ATR mode showed characteristic wave numbers (*ν*∼) to the functional group bonds associated with the pigment chemical structure (Fig. 4). According to the *ν*∼ values we have been identified the frequency of: symmetric and asymmetric vibration of C–O–C at 1100–965 cm^−1^, the stretching of C–N bond at 1214 cm^−1^ and the C=C bond at 1600 cm^−1^. The IR spectrum showed the presence of carboxyl functional groups, based on two related vibrations: –C(=O)–OH at 1700 cm^−1^ and for the asymmetrical and symmetrical stretching of the C=O at *ν*∼ between 2830 - 2695 cm^−1^. While for the C–H bond frequency is at *ν*∼ 2900 cm^−1^ and the range of *ν*∼ 3500–3000 cm^−1^ represents the stretching vibration of the –OH functional group.^18,19^ The corresponding frequencies of the alkanes, hydroxyls, alcohols, carboxylic acids and esters bonds in the graph show similar to those reported in β-d-Glucose studies.^20–22^ It suggests that the pigment has a glucoside part. The presence of the carboxylic groups reinforces the idea of the electrostatic interaction between pigments and cations. Comparing to betalain IR spectrum, the results share OH and C–O–C vibration band^23^ However, it is not enough to determine a pigment structure, thus further characterization were done.

**Fig. 5:**
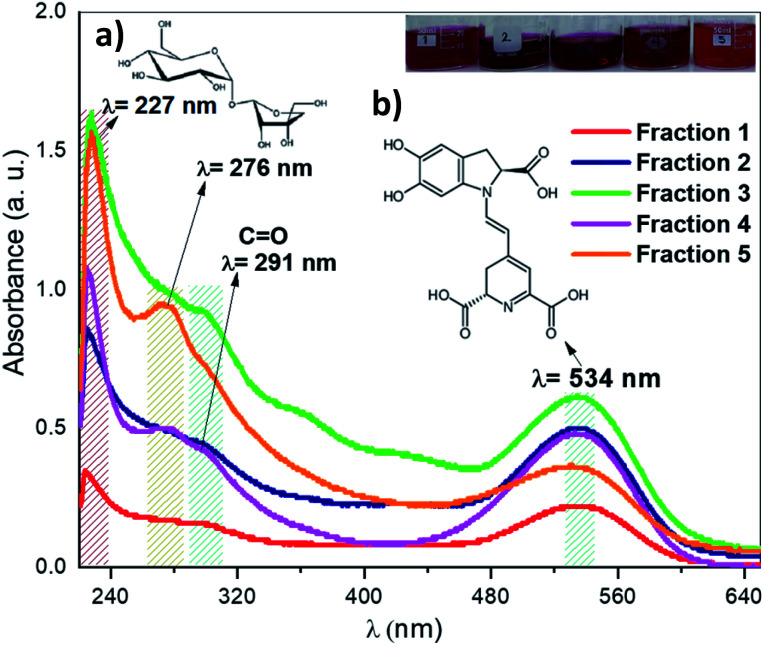
UV-vis spectroscopy of the colored fractions obtained from ayrampo extract by chromatography column. Region corresponding to (a) glucose molecule and apiose at 227 nm and 276 nm, respectively. (b) Region corresponding to betanin pigment at 534 nm.

After the purification process of the pigments from ayrampo extract through silica gel chromatography column, we performed UV-vis spectroscopy to identify the photoluminescent fractions. Fig. 5 shows the UV-vis spectra of the five fractions obtained during the initial pigment purification process. We found the presence of two absorption peaks at 227 nm and 276 nm, which correspond to the glucose and apiose, respectively (region 5a).^24^ From these results all fractions probably have both molecules linked to each other by an oxygen bond through condensation reaction. As well the absorption peak of at 534 nm (region 5b) confirms the betalain core structure.^25–27^ Results show also a peak at ≈ 291 nm corresponding to the characteristic C=O bond of the carboxylic group, which suggests the presence of crotonic acid.^28–32^ From these data, we calculate the concentration of betalain in each fraction using the equation *A* = *ε* × *l* × *c*. Where, A is the absorbance, *ε* is the wavelength-dependent molar absorptivity coefficient, *l* is the length of the cuvette and *c* is the concentration of the solution. The results show that fraction 3 has the highest concentration of betalain, value equal to 10 μmol L^−1^ using the molar absorption data *ε*_538 nm_ = 60 000 M^−1^ cm^−1^ in H_2_O, MW = 550 g mol^−1^.^33^ The absorbance of this one decreases in fraction 1, corresponding to a betalain concentration of 3.8 μmol L^−1^. Through this characterization, we can rule out that the molecules of cyclo-DOPA and betaxanthin are separated in the solution. Because, if they are separated, it should show a strong absorption in the blue region, with molar absorptivity *ε* = 45 000 M^−1^ cm^−1^ at 470 nm and 500 nm.^11,27^ However, it is not the case, the pigment absorbs in the green region showing that betalain is part of the composition. On the other hand, fraction 5 has a concentration of ≈ 6 μmol L^−1^ and exhibits well-defined absorption peaks corresponding to the characteristic functional groups of the pigment. Thus, this fraction was selected to perform further extraction steps, in order to make it possible, we mixed fraction 5 with hydrated acetone (CH_3_–CO–CH_3_ : H_2_O, 8 : 1 ratio) at pH = 4.6, then it was taken to a silica gel chromatography column and the main fraction was analysed by mass spectroscopy.

**Fig. 6:**
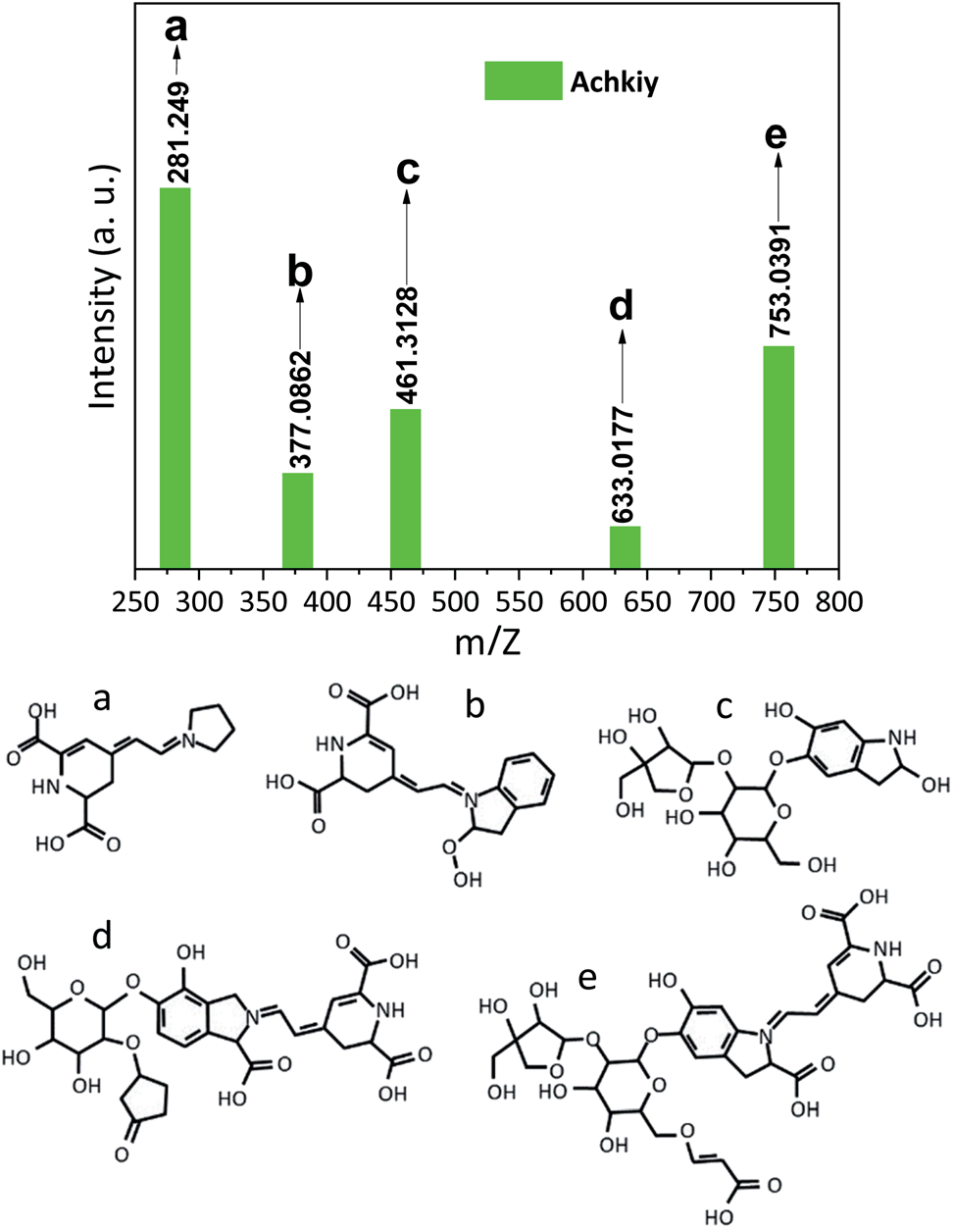
Mass spectrophotometry of the pigment showing particular ionizing fragments from a to e.

The structure of the molecule contained in the selected fraction was finally identified by the Electron Spray Ionization (ESI) signals. Fig. 6 shows five fragments. The fragment (6a) is the most abundant specie *m*/*z* [M + H^+^] = 281.249 which corresponds to the molecule formed by the reaction of betalamic acid and a pyrrole in acid condition.^22^ The formation of the different ionic species during the analysis of MS shows the cyclo-DOPA molecule bound to the structure of betalamic acid (6b) *m*/*z* [M + H^+^] = 377.0862. However, the presence of *m*/*z* [M + H^+^] = 461.3128 of the apiosyl species is only detected bound to carbon five of the cyclo-DOPA (6c).^34^ While, the structure of the betanin bound to 5 carbon ring (6d) has *m*/*z* [M + H^+^] = 633.0177. Finally, the larger fragment is present at (6e), which corresponds to the betanin structure bounded to apiosyl radical and crotonic acid on the glucoside region *m*/*z* [M + H^+^] = 753.0391.^35^ We called this fragment Achkiy.

Then, we determined the Molecular Lipophilic Potential (MLP) on van der Waals Surfaces^36^ by calculation of the Achkiy molecule. The calculated logP value was −5.83 with a volume of 614.82 Å^3^. This value corresponds to a hydrophilicity compound and shows high correlation of the molecule with hydroxyl groups presence hydrophilicity.

**Fig. 7:**
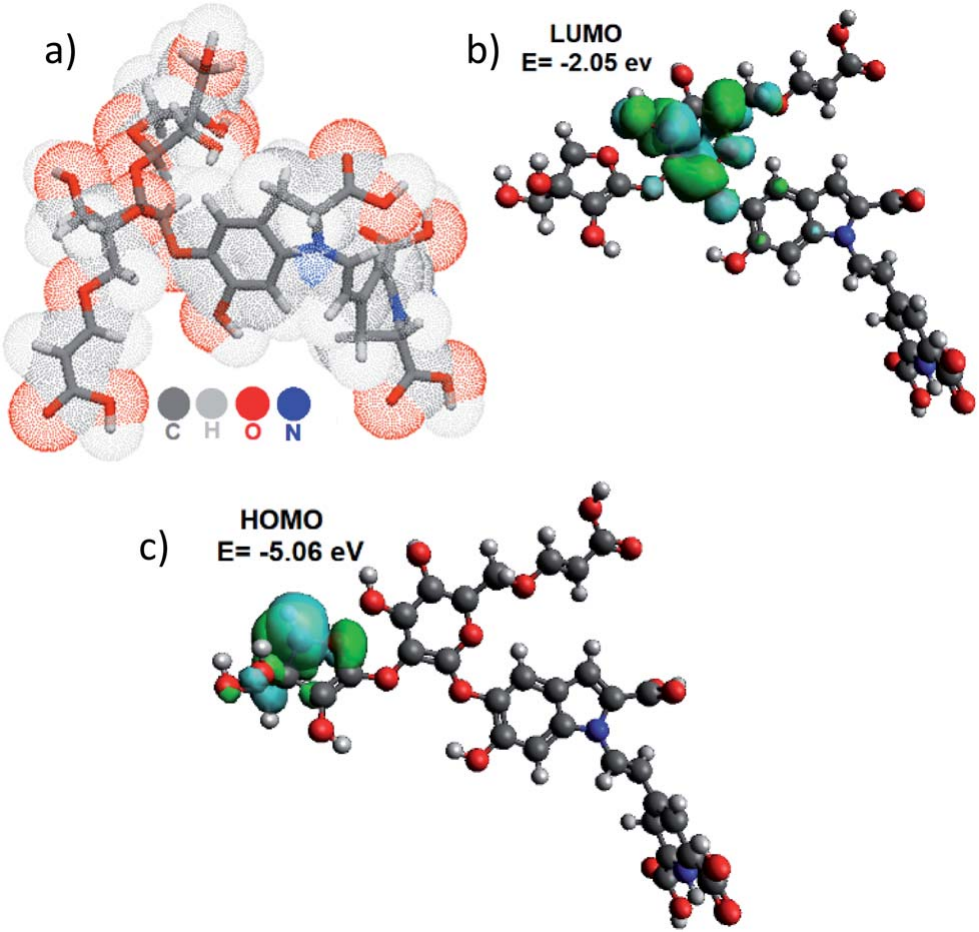
(a) Molecular Lipophilic Potential for the 3D structure of the Achkiy molecule at vacuum condition and (b) LUMO and (c) HOMO.

Fig. 7 a shows the MLP graph to see which part of the surface are hydrophobic (coded by blue color) and which are hydrophilic (red). This result suggests that the pigment is dissolved in a polar substance, and has a high permeability (10^−5.83^). On the other hand, we optimized the Achkiy molecule and calculated the HOMO–LUMO gap value (3.01 eV), which differs from the betanin (1.54 eV).^8,37^ Thus, it requires more energy to create excitons, blue shift absorption. Fig. 7b and c show the localization of the HOMO and LUMO of the Achkiy molecule, giving an idea about the charge transfer among the functional groups. Since the HOMO is located onto apiosyl and LUMO on cyclo-DOPA, we suggest that the charge transfer between the moieties occurs through the transition π → π*. From these results, we identify the Achkiy as a molecule with donor–acceptor intramolecular charge transfer (D–A–π–A), constructed by electron-rich (apiosyl) and electron-deficient (cyclo-DOPA) moieties.^5^

**Fig. 8:**
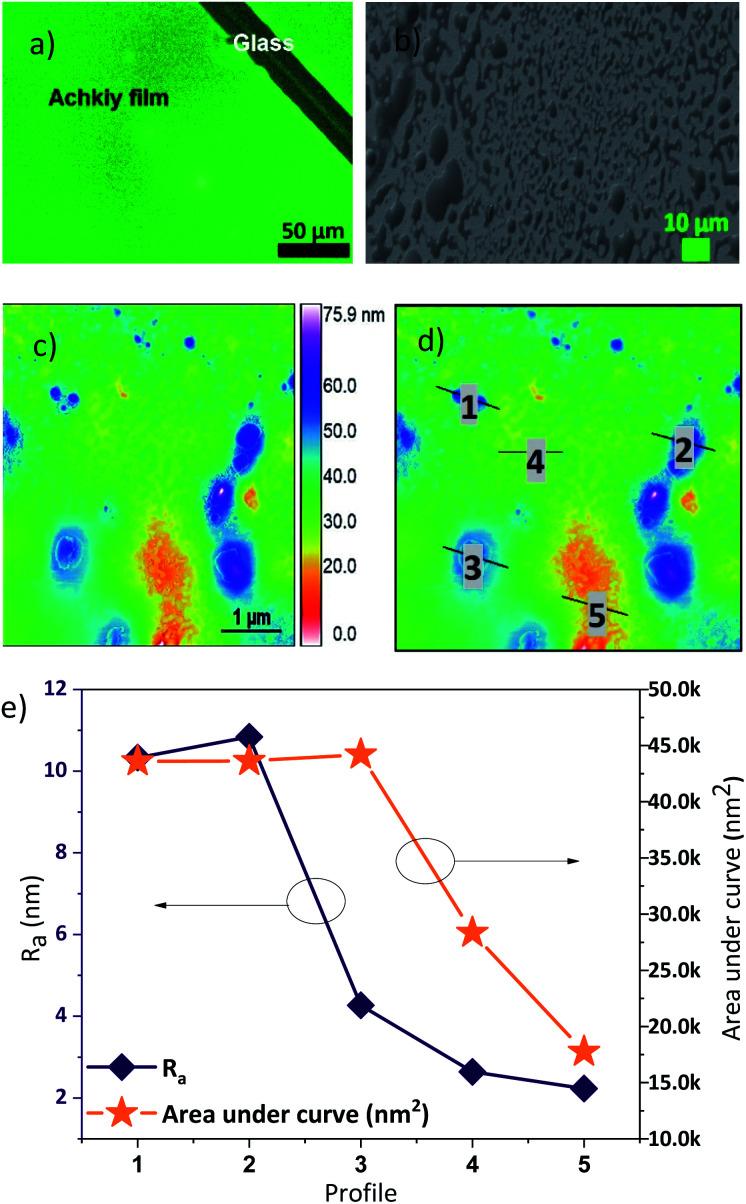
(a) Fluorescence, (b) SEM and (c) AFM images of Achkiy thin film, showing the topography of the photoluminescent film. (d) Profiles analysed and (e) parameters of *R*_a_ for each one of them.

In order to build an OLED device we characterized first the Achkiy film onto electrode Indium Tin Oxide (ITO). Fig. 8 shows the fluorescence microscopy of the Achkiy thin film surface and the characterization of it using SEM and Atomic Force microscopy (AFM). Fig. 8a displays a homogeneous photoluminescent surface when the film is irradiated with a *λ* = 380 nm. Then, we evaluated the morphology of the surface by SEM microscopy. It showed that this film has a surface with micro clusters that oscillate between 0.5 and 20 μm in diameter (Fig. 8b). While, the roughness of the thin film (*R*_a_) reached a value of ≈ 75.9 nm (Fig. 8c). For more details of the film, we selected five profiles according to their *R*_a_ value, which are depicted in Fig. 8d. These longitudinal profiles are shown in Fig. 8e and these were analysed considering the higher and lower *R*_a_ and area values. The higher value is the profile 2 (with roughness, *R*_a_ ≈ 10.84 nm) which shows a condensation of Achkiy. It could be related to the β-d-Glucose molecules,^31,32^ since the Achkiy presents a caramelized viscous texture after the drying process and its profile has an area ≈ 43 623 nm^2^. While the lower profiles 4 and 5 with values of *R*_a_ ≈ 2.64 nm and *R*_a_ ≈ 2.23 nm respectively, show almost flat surfaces with area values equal to 28 344 nm^2^ and 17 751 nm^2^.

**Fig. 9:**
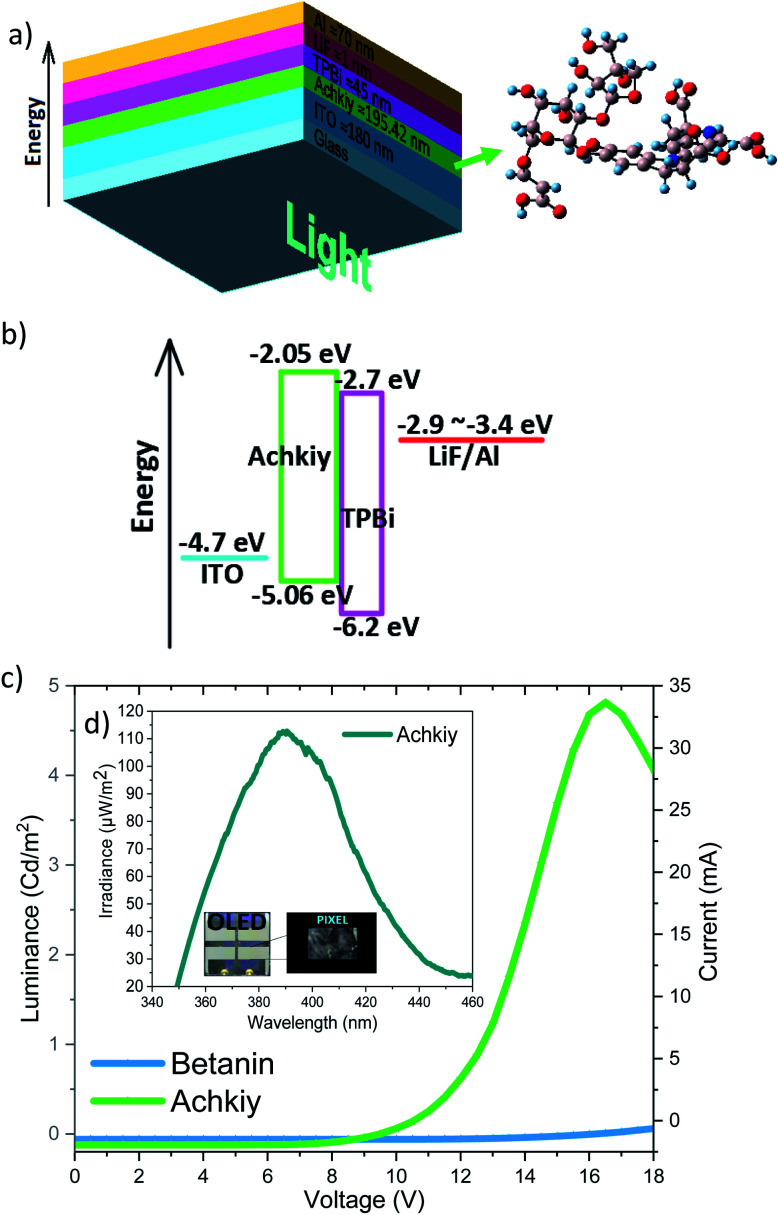
(a) OLED sandwich structure and thicknesses of each thin layers. (b) The bang gap energy (eV). (c) Voltage curve (V), current (mA) and luminance (Cd m^−2^) of the Achkiy and Betanin OLED. (d) Device irradiance curve.

We evaluated the luminance efficiency of the Achkiy-based OLED with the following flat architecture: glass/ITO/Achkiy/TPBi/LiF/Aluminium (Fig. 9a). Each one of these layers have a band gap (Fig. 9b) and their arrangement allows the charge flow among of them. This OLED shows a luminance of 4.8 Cd m^−2^ by applying a bias voltage ≈ 16.5 V and a current ≈ 34.1 mA (Fig. 9c) while the irradiance reached 113.3 μW m^−2^ (Fig. 9c, d). In order to compare the luminescence efficiency of the Achkiy layer within an OLED, we manufactured one based on betanin (with same conditions and parameters), resulting in a zero electrical reaction when a voltage was applied. This applied voltage causes the charges to move due to the electric field allowing the hollows and excited electrons be replaced by ITO and TPBi, respectively. Thus, after electrons and holes are transported to the emitting layer, opposite charges recombine to generate excitons. We suggest that the recombination occurs because the electrons from TPBi flows through the LUMO, forming a transporting channel through the oxygen bridge^38,39^ to following their travel through the π–π stacking interactions of the molecule to ITO interface. The charges are injected into the ITO^40^–Achkiy interface due the presence of the oxygen functional anchoring at the crotonic acid of the organic molecule (2′-*O*-Apiosyl-6′-*O*-crotonic acid). It allows the hollows transportation. However, the charges recombination is unfavourable in betanin based OLED because it lacks the long carboxylic group appendix. The Achkiy presents an interesting response in OLED and requires more studies in order to improve the thin layer production. Since, the clusters of the Achkiy thin film mean that light is not generated homogeneously throughout the pixel area. Causing that the low consumption of electric current of this device, reaching an electrical work of 0.56 Watts.

**Fig. 10:**
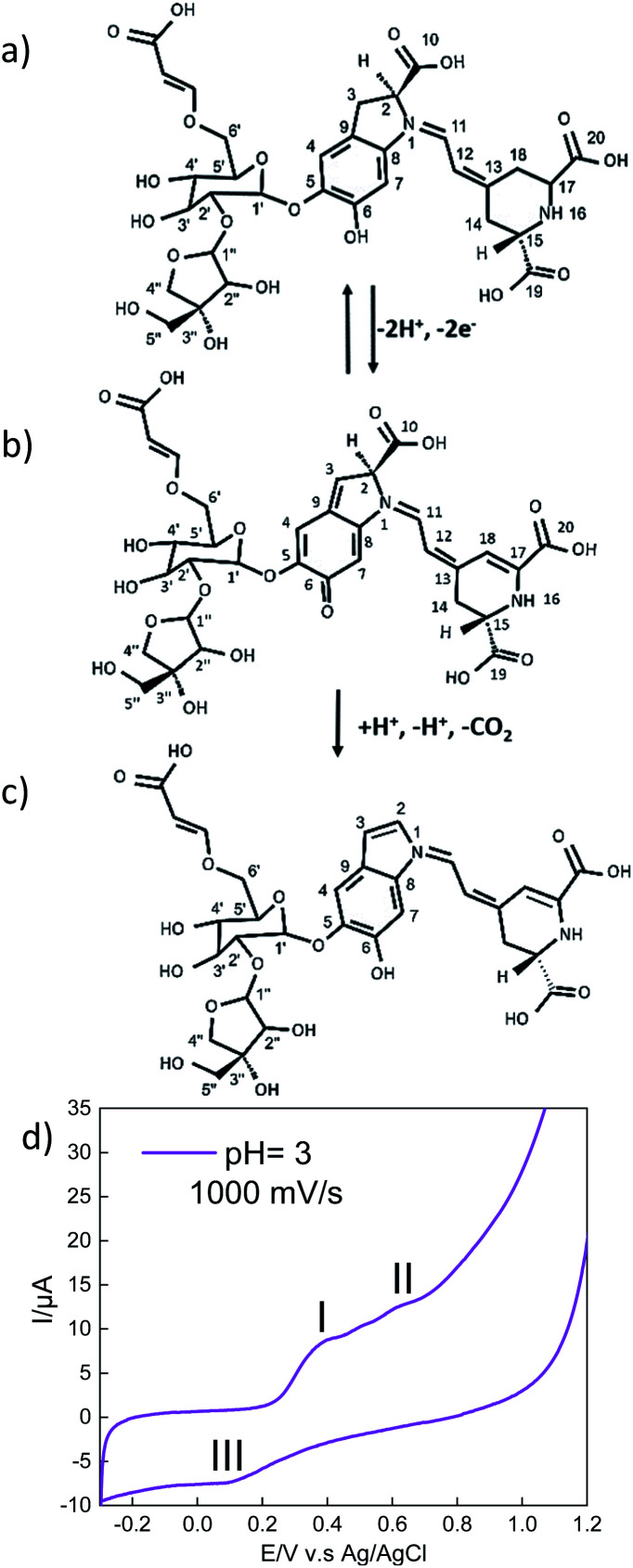
Proposed reaction pathways on (a) Achkiy molecule. (b) First deprotonation at oxygen 6 and carbon 3. (c) Second deprotonation and decarboxylation at carbon 10. (d) Achkiy cyclic voltammetry on a glassy carbon electrode (1.8 mm in diameter) obtained in a phosphate-acetic buffer medium at a pH = 4.6 and scanning speed of 1000 mV s^−1^ and at different potentials.

Another important characteristic of this emissive layer type is the stability toward free radicals when the device is turn on, which is closely related to the electron donation ability of betalains.^41^ It is relevant because the Achkiy thin layer shows a REDOX potential (Fig. 10). In this sense, Slimen *et al.*^11^ reported that betalains stabilize radicals due to the presence of dihydropyridine ring. Thus, we suggest that the Achkiy bring stability to the OLED because its structure shares the same betalain structure backbone. Since the process of light emission is due to the oxidation of the Achkiy molecule (Fig. 10a). We suggest the possible electron flow (Fig. 10b, c) based on the cyclic voltammetry results. Fig. 10d shows the cyclic voltammetry of the Achkiy solution using a carbon glassy electrode, in which we confirmed the two oxidative stages (−2e^−^) at the anode: (I) *I*_pa_ = 8.49 μA, *E*_pa_ = 0.38 V and (II) *I*_pa_ = 12.5 μA, *E*_pa_ = 0.62 V; while the reduction stage (III) occurs in the cathode: *I*_pc_ = −7.31 μA, *E*_pc_ = 0.09 V. The process of two electrons, one proton is probably attributed to the low pH. Moreover, the possible Achkiy oxidation process path could generate a transient radical cation. This transient compounds rapidly loses a proton at oxygen 6 and carbon 3 (I), which leads to a neutral phenoxy radical. (II) shows the second suggested oxidation producing decarboxylation in the molecule (at carbon 10), the loss of the second proton occurs at cyclo-DOPA (–COO^−^). In overall, it results in the formation of an oxidized species of two electrons based on hydroquinone structure moiety when the device is turn-on-voltage. And in according to the arrangement of layers bandgap, we suggest the hydroquinone moiety recovery occurs when the device is turned off, causing the electrons to decay to a lower level in the TPBi LUMO.^11,41^ The electronic decay, when the devices is turn on, produces a photon with a *hv* ≈ 390.12 nm (blue region).

The charges recovering occurs during the polarization process due to the applied potential (16.5 V) when the cycle process is closed in the Achkiy based OLED. Furthermore, through the cyclic voltammetry of Achkiy, it can be confirmed that part of its reduction – oxidation process is involved by the interaction of its oxygenated functional groups by participating in the process the *cis* and *trans* bonds due to the flow of electrons.

## Conclusions

To conclude, the Achkiy extracted from the ayrampo pigment when diluted in hydrated acetone showed an electroactive activity by applying a DC voltage of 16.5 V and a current of 34.1 mA causing the OLED to emit light with a *λ* = 390.12 nm. This process involved the transport of holes from the ITO and the injection of electrons from the TPBi into the LUMO of the molecule, moved by the electric field generated by the polarization voltage applied to the device. In addition, it is important to emphasize that Apiosyl-6′-*O*-crotonic acid possibly recombine the charges due to the function of anchoring through –OH bridges at the interface between ITO and Achkiy. Furthermore, Achkiy is shown as a molecule with a broad field of research in the optoelectronics of eco molecules, where its extraction process involves a green chemical process and where the main solvents used due to its high polarity water, ketone and citric acid makes the Achkiy molecule acquire interesting physicochemical properties for the field of bio-electroluminescence and is necessary to improve the Achkiy thin film layer quality in order to increase the OLED luminescence efficiency.

## Conflicts of interest

The authors declare no competing financial interest.

## Supplementary Material
